# Newborn Body Fat: Associations with Maternal Metabolic State and Placental Size

**DOI:** 10.1371/journal.pone.0057467

**Published:** 2013-02-27

**Authors:** Camilla M. Friis, Elisabeth Qvigstad, Marie Cecilie Paasche Roland, Kristin Godang, Nanna Voldner, Jens Bollerslev, Tore Henriksen

**Affiliations:** 1 Section for Obstetrics, Women and Children’s Division, Rikshospitalet, Oslo University Hospital, Oslo, Norway; 2 Outpatient Clinic for Specialized Endocrinology, Division of Medicine, Rikshospitalet, Oslo University Hospital, Oslo, Norway; 3 Faculty of Medicine, University of Oslo, Oslo, Norway; VU University Medical Center, The Netherlands

## Abstract

**Background:**

Neonatal body composition has implications for the health of the newborn both in short and long term perspective. The objective of the current study was first to explore the association between maternal BMI and metabolic parameters associated with BMI and neonatal percentage body fat and to determine to which extent any associations were modified if adjusting for placental weight. Secondly, we examined the relations between maternal metabolic parameters associated with BMI and placental weight.

**Methods:**

The present work was performed in a subcohort (n = 207) of the STORK study, an observational, prospective study on the determinants of fetal growth and birthweight in healthy pregnancies at Oslo University Hospital, Norway. Fasting glucose, insulin, triglycerides, free fatty acids, HDL- and total cholesterol were measured at week 30–32. Newborn body composition was determined by Dual-Energy X-Ray Absorptiometry (DXA). Placenta was weighed at birth. Linear regression models were used with newborn fat percentage and placental weight as main outcomes.

**Results:**

Maternal BMI, fasting glucose and gestational age were independently associated with neonatal fat percentage. However, if placental weight was introduced as a covariate, only placental weight and gestational age remained significant. In the univariate model, the determinants of placenta weight included BMI, insulin, triglycerides, total- and HDL-cholesterol (negatively), gestational weight gain and parity. In the multivariable model, BMI, total cholesterol HDL-cholesterol, gestational weight gain and parity remained independent covariates.

**Conclusion:**

Maternal BMI and fasting glucose were independently associated with newborn percentage fat. This effect disappeared by introducing placental weight as a covariate. Several metabolic factors associated with maternal BMI were associated with placental weight, but not with neonatal body fat. Our findings are consistent with a concept that the effects of maternal BMI and a number of BMI-related metabolic factors on fetal fat accretion to a significant extent act by modifying placental weight.

## Introduction

Determinants of birthweight and neonatal body composition have gained increasing attention over the recent years. This interest has largely been driven by the well documented relation between anthropometric characteristics at birth and future health of the newborn [1]. In addition fetuses of small or large size are at increased risk of perinatal complications [2]. However, birthweight is only a crude measure of intrauterine growth and especially of neonatal body composition. Growth of the human fetus involves a characteristic accretion of adipose tissue in the last trimester. Neonatal body fat is therefore an important indicator of fetal energy supply and growth conditions [3], [4].

Maternal body mass index (BMI) is one of the most consistent determinants of fetal weight, growth and body composition [4], [5]. However, BMI is not a biological effector, and the mechanisms by which BMI exerts its effects on fetal growth and body composition remain largely unknown [6]. In general, besides placental endocrine functions, the factors governing the growth of a healthy fetus may be divided into three groups; the genetic potential of growth, maternal supply of nutrients and the capacity of the placenta to transfer nutrients from the maternal circulation to the fetus. In particular, maternal BMI may affect fetal growth and fat mass accretion both by modifying the supply of nutrients and by affecting placental nutrient transport capacity.

Maternal BMI is positively associated with circulating glucose. Furthermore, maternal BMI and glucose are established as independent determinants of large for gestational age newborns and excessive body fat at birth [7], [8]. Consequently, other factors than glucose is likely to play a role in the association between BMI and fetal fat mass accretion [7], [9], [10]. Studies of the effects of other metabolic factors linked to increasing BMI, like lipids (cholesterol, triglycerides and free fatty acids) and insulin on fetal growth are inconsistent [11], [12].

While numerous studies have identified maternal nutritional and metabolic measures as determinants of fetal growth, fewer have evaluated the role of placental transport capacity in this relation [13], [14]. Placenta has however, a pivotal role in fetal growth as all nutrients need to pass the syncytio-vascular layer of placenta in order to enter fetal circulation. Maternal BMI has been shown to affect both placental properties and weight, and placental weight is closely associated with fetal growth [15], [16]. Placenta is furthermore believed to act as a nutrient sensor, up- or down regulating transport proteins according to the maternal environment [13]. Thus it is conceivable that some of the metabolic derangements associated with increasing BMI play a role in regulating placental function. There is no single parameter reflecting placental function, but placental transport capacity is dependent on exchange area as well as density of transport proteins. Under physiological conditions, it is reasonable to assume that placental weight is correlated to exchange area and therefore with total transport capacity [17], [18].

The objective of the current study was first to explore the relationships between maternal nutritional and metabolic status (as reflected in maternal BMI, plasma levels of glucose, insulin, triglycerides, free fatty acids and cholesterol) on neonatal percentage body fat. Then we wanted to determine to which extent any associations were modified when considering placental size in these relations. Finally, we examined if maternal metabolic parameters associated with BMI were associated with placental weight.

## Materials and Methods

The present work was performed in a subcohort (n = 207) of the STORK study, an observational, prospective study on the determinants of fetal growth and birth weight in healthy pregnancies (details have been published previously) [19], [20]. In short, the STORK-cohort consists of women of Scandinavian heritage (n = 1031) who registered for obstetric care at Oslo University Hospital - Rikshospitalet from 2001–2008. Exclusion criteria were multiple pregnancies, known pre-gestational diabetes, and severe chronic diseases (lung, cardiac, gastrointestinal or renal).

The women were scheduled for four prenatal visits (visit 1–4) at gestational weeks 14–16, 22–24, 30–32 and 36–38. Gestational age was based on ultrasound measures made at weeks 17–19. Data on age, parity, educational level, smoking status and pregestational BMI were obtained by interview. Data on preeclampsia and hypertension were obtained from hospital records.

Maternal height was obtained at the first visit and weight was measured by a calibrated scale at each visit. Maternal BMI (kg/m^2^) was then calculated based on first visit measures. Oral glucose tolerance test (75 g –2 hour) was undertaken twice, at weeks 14–16 and 30–32. Fasting plasma glucose was measured by Accucheck (Roche Diagnostics, Mannheim, Germany).

Fasting blood samples were collected in 7-ml Vacutainer tubes, centrifuged at room temperature at 3000 RCF in 10 min, aliquoted and stored at −80°C until analyzed. Samples for insulin were assayed in duplicate (RIA, DPC, Los Angeles, CA), and the intra- and interassay CV were 4.9 and 5.4%, respectively. Total cholesterol, high density lipoprotein cholesterol (HDL cholesterol) and triglyceride assays were performed by accredited laboratories according to standard laboratory methods (Department of Medical Biochemistry, Oslo University Hospital Rikshospitalet, Oslo). The concentration of free fatty acids (FFA) was measured by a NEFA (nonesterified free fatty acid) C-kit, WAKO Chemicals (Neuss, Germany), based on an enzymatic colour reaction following activation and oxidation of the free fatty acid. Intra-assay variation was <6.0%.

Placentas, including cord and membranes were weighed and gross placental pathology registered within 1 hour after delivery.

Newborn body composition was determined by Dual-Energy X-Ray Absorptiometry (DXA) (GE Lunar Prodigy densitometer (software version 12.10; GE Medical Systems, Lunar Corp., Madison,WI)). All DXA scans were performed within 4 days postpartum. The scanning procedure has been described in detail previously [19].

DXA measurements provided information on bone mass, total fat mass and fat-free mass. Percentage fat was based on DXA-derived fat ÷ DXA-derived sum of fat, lean and bone mineral content.

Written informed consent was obtained from the participants. The study was approved by the Regional ethics committee, Southern Norway.

### Statistical Methods

Data are presented as mean and standard deviation (SD), frequency and percentage (%), or median and interquartile ranges (Q1, Q3). We log transformed insulin to obtain a normal distribution and excluded values above 3 SD from mean (one value).

Linearity in bivariate associations was assessed by Loess smoothing curves. Bivariate associations were explored using Pearson correlation coefficients and then tested for collinearity (r_p_>0.7). The associations were further explored by linear regression models, both univariate and multiple linear regression models. All analyses with birthweight, neonatal fat percentage and placental weight as the outcome variable were adjusted for gestational age.

The outcome measures were newborn percentage fat, birthweight and placental weight. The determinants included maternal BMI, weight gain, parity, age, fasting glucose, lipids (free fatty acids, triglycerides, HDL- and total-cholesterol) and insulin.

In multiple linear regression models all covariates with P-values <0.1 in the univariate analysis were included.

Placental weight was considered as a determinant of neonatal fat percentage and two multiple regression models were fitted; one which included maternal BMI, glucose, and gestational age, and one which included the same covariates plus placental weight.

The ratio between birth weight and placenta weight is considered as an indicator of placental efficacy [21]. Therefore, besides studying determinants of placenta weight we assessed determinants of the feto-placental weight ratio.

All p-values <0.05 were considered statistically significant.

Statistical analysis was performed using the Statistical Package for Social Sciences (SPSS, Version 18.0) for Windows (SPSS Inc., Chicago, IL).

## Results

The demographic and clinical details of the cohort are presented in Table 1 (Maternal characteristics) and Table 2 (Infant characteristics).

**Table 1:** Clinical and demographic characteristics of the mothers.

Maternal characteristics	Percentage or Mean (SD)
Higher Education (>12 years)	86.5%
Primipara	44%
Smokers (daily)	1%
Age	31 (3.5)
Pre pregnancy Height (cm)/weight (kg)	168/66
GWG (kg)***	10.6 (3.1)
BMI (kg/m^2^) *	23.9 (3.7)
Fasting glucose (mmol/l) **	4.0 (0.4)
Insulin (pmol/l) **	43 (25)
Triglycerides (mmol/l)**	2.01 (0.65)
Free fatty acids (mmol/l) **	0.44 (0.13)
Cholesterol – total (mmol/l)**	6.96 (1.2)
Cholesterol – HDL (mmol/l)**	1.71 (0.37)
Blood pressure (visit 1) mmHg*	111/68 (9/8)
Placental weight (g)	703 (161)
Complications:	
Hypertension/preeclampsia#	7/5 (3.4%/2.4%)
GDM (WHO)##	9 (4.3%)

* Visit 1 (weeks 14–16).

** Visit 3 (weeks 30–32).

*** Gestational weight gain from 14–16 to 36–38 weeks.

# Blood pressure ≥140/90 with/without proteinuria (≥1+ on dipstick).

## Gestational diabetes based on WHO criteria (75 g oral glucose tolerance test and corresponding 2 h- glucose value ≥7.8 mmol/l).

**Table 2:** Clinical characteristics of the infants.

Infant characteristics	Percentage or Mean (SD)
Birthweight (g)	3592 (492)
Gestational age at birth	40.1 (1.4)
Gender	46%girls/54% boys
**DXA:**	
Scan day (after birth)	1.8 (0.9)
Scan weight, DXA estimation (g)	3408 (473)
Fat mass (%)	13.6 (2.6)
Fat mass (g)	494 (123)

DXA; Dual Energy X-Ray Absorptiometry.

The cohort consisted of 207 mother –infant pairs. Mean maternal age was 31 years, 44% were primipara and maternal BMI ranged from 17–39 kg/m^2^ with a mean of 24 kg/m^2^.

The study population was comparable to women who gave birth in Oslo on parameters including birthweight and maternal age, and placental weight and birthweight-placental weight ratio were also comparable to those reported in a Norwegian percentile curves study [22].

Mean birthweight was 3592 g and mean gestational age at birth was 40 weeks. Neonatal fat percentage ranged from 8.3% to 19.6% with a mean of 13.6%. Newborn girls had a significant higher mean fat percentage than boys (14.4% versus 12.7%). Table 3 shows the correlations (Pearson correlation coefficients) between maternal BMI and metabolic measures.

**Table 3:** Correlations between maternal BMI and metabolic parameters (Pearson correlation coefficient).

	BMI	Glucose	Insulin (ln)	Cholesterol-total	Cholesterol-HDL	Triglycerides	Free fatty acids
BMI	1						
Glucose	0,36*	1					
Insulin (ln)	0,54*	0,43*	1				
Cholesterol-total	ns	ns	ns	1			
Cholesterol-HDL	−0,20*	−0,23*	−0,24*	0,15*	1		
Triglycerides	0,27*	0,24*	0,38*	0,24*	−0,49*	1	
Free fatty acids	0,18*	ns	ns	ns	−0,18*	0,32*	1

* Significant correlation, p<0.05.

In univariate analysis, maternal BMI (β = 0.13), fasting glucose (β = 1.2) and HDL-cholesterol (β = −0.85) were all significant (p<0.003) determinants of *neonatal fat percentage*. None of the other metabolic measures, age, parity or gestational weight gain was significantly related to fat percentage. In contrast, triglycerides, weight gain, parity and maternal age were all significant determinants of *birthweight*, besides maternal BMI, fasting glucose and HDL-cholesterol (Table 4).

**Table 4:** Maternal determinants of birth weight and percentage neonatal body fat.

	Birthweight		Neonatal percentage fat		
Determinants	Univariate*Unadjusted B (CI)	MultipleAdjusted B (CI)	Univariate*Unadjusted B (CI)	Multiple Model 1Adjusted B (CI)	Multiple Model 2Adjusted B (CI)
Gestational age**	169 (126, 212) p<0.001	154 (112, 196) p<0.001	0.35 (0.12, 0.58) p = 0.004	0.34 (0.12, 0.20) p = 0.004	0.26 (0.04, 0.5) p = 0.03
BMI***	31 (15,46) p<0.001	22 (7,38) p = 0.006	0.13 (0.04,0.22) p = 0.003	0.098 (0.01, 0.19) p = 0.036	0.06 (−0.03, 16) p = 0.189
Fasting glucose	250 (111,389) p<0.001	150 (5,296) p = 0.043	1.2 (0.48, 2.0) p = 0.002	0.84 (0.08, 1.67) p = 0.048	0.76 (−0.07, 1.6) p = 0.071
Placental weight			0.004 (0.002, 0.006) p<0.001		0.003 (0.001, 0.005) p = 0.005
Insulin	ns		ns		
Free fatty acids	ns		2.4 (−0.04, 4.9) p = 0.054	ns	
Triglycerides	94 (2,187) p = 0.046		ns		
Cholesterol (total)	ns		ns		
Cholesterol (HDL)	−170 (−329, −9) p = 0.04		−0.85 (−1.7, −0.002)p = 0.049	ns	
HDL/tot.chol ratio	ns		ns		
GWG****	29 (10,48) p = 0.003	34 (16,52) p<0.001	ns		
Parity	241 (124, 358) p<0.001	210 (97,322) p<0.001	ns		
Age	19 (2.6, 37) p = 0.024		ns		

Results from univariate and multiple linear regression.

* Adjusted for gestational age at birth.

** Gestational age at birth.

*** BMI (kg/m^2^) was measured in gestational week 14–16.

**** Gestational weight gain 14–16 to 36–38 weeks.

Fasting glucose, insulin, free fatty acids, triglycerides and cholesterol (mmol/l) were measured at gestational week 30–32.

In multiple regression analysis of neonatal fat percentage as the outcome, HDL and FFA were included, in addition to BMI, gestational age and glucose. In this model, gestational age, maternal BMI and glucose remained independent covariates.

Using birthweight as the dependent variable, the same covariates (gestational age, BMI and glucose) were significant, together with parity and weight gain (Table 4).

Secondly, we considered the role of placental weight in relation to neonatal percentage fat. In univariate analysis, placental weight was a significant determinant of fat percentage (p<0.001), and explained approximately 10 percent of the variation (R^2^ = 0.098). If placental weight was adjusted for in a multiple model with neonatal percentage fat as dependent variable (model 2), only gestational age and placental weight remained significant, while BMI and glucose became non significant (Table 4).

We then explored possible maternal determinants of placental weight. In univariate analysis, gestational age, BMI, insulin, triglycerides, HDL- cholesterol (negatively), total cholesterol and HDL/total cholesterol ratio were all significant determinants of placental weight. Glucose was borderline significant (p = 0.052) while the level of fatty acids was not significantly associated with placental weight. In addition, gestational weight gain (p = 0.003) and parity (p = 0.02), but not maternal age, were significant determinants of placental weight. In a multiple model, all covariates except free fatty acids were included. Gestational age, gestational weight gain (GWG), parity, maternal BMI, total cholesterol and HDL cholesterol remained independent determinants of placental weight.

Finally, we considered birthweight-placental weight ratio, an indicator of placental efficacy, as the outcome [23]. We found a negative association between maternal BMI (β = −0.46, p = 0.01), insulin, total cholesterol, triglycerides and birthweight-placental weight ratio. Glucose, parity, age, weight gain and HDL were not associated with placental efficacy (Table 5).

**Table 5:** Maternal determinants of placental weight.

	Placental weight			
Determinants	Univariate regression *		Multiple regression **	
	Unadjusted B (95%CI)	p-value	Adjusted B (95%CI)	p-value
Gestational age	29 (13,44)	p<0.001	26 (10,42)	p = 0.001
BMI	12 (7,18)	p<0.001	10 (5, 16)	p = 0.005
Glucose	51 (−0.4, 103)	p = 0.052		
Insulin	1.2 (0.4,2.1)	p = 0.005		
Free fatty acids	66 (−104,236)	p = 0.45		
Triglycerides	55 (22,88)	p = 0.001		
Cholesterol (tot)	18 (0.06,36)	p = 0.049	25 (8,42)	p = 0.004
Cholesterol (HDL)	−71 (−129, −13)	p = 0.016	−60 (−115, −5)	p = 0.034
HDL/tot chol	−509 (−838, −180)	p = 0.003	Not included	
GWG ***	11 (6,18)	p = 0.003	13 (6,19)	p<0.001
Parity (0,more than 1)	52 (9,96)	p = 0.018	48 (6,89)	p = 0.025
Maternal age	ns			

Results from univariate and multiple linear regression.

* Adjusted for gestational age.

** Adjusted for gestational age and all listed determinants of placental weight except free fatty acids.

*** Gestational weight gain 14–16 to 36–38 weeks.

## Discussion

In the current study we found that maternal BMI and fasting glucose are associated with neonatal fat percentage, in accordance with the HAPO-study [8]. However, these effects were found to be markedly modified, in fact no longer significant, if adjustment for placental weight was allowed.

Secondly, we found that several of the metabolic parameters associated with BMI, including total cholesterol and HDL- cholesterol were related to placental size.

Thus, the maternal factors that were associated with percentage neonatal body fat differed from those that were associated with placental weight.

Maternal BMI is one of the most consistent determinants of fetal growth in observational studies, irrespective of how fetal growth is evaluated, being it birthweight, large for gestational age (LGA), accelerated fetal growth, increased abdominal circumference, neonatal caliper measurements or fat percentage [3], [8], [24]–[26]. Neonatal adiposity may be considered a more appropriate indicator of surplus energy supply to the fetus than birthweight as increased neonatal fat may also be present in normal weight neonates [2], [3]. Furthermore, neonatal fat mass is believed to better reflect intrauterine environment and energy supply in the last trimester, whereas lean mass is thought to be more subjected to genetic influences [26].

Our finding that maternal BMI was significantly associated with neonatal fat percentage (Model 1 Table 4) is thus in line with previous reports. Although our results confirm that weight gain and parity are independent determinants of birthweight, we found no evidence for an effect of weight gain and parity on fetal fat accretion. These differentiated effects illustrate that birth weight and neonatal adiposity are not identical entities, and are likely to reflect different aspects of fetal growth.

BMI is not a biological effector. BMI relies on weight and height and is a substitute measure of the state of adiposity. In the non-pregnant population BMI, insulin resistance, hyperglycemia, dyslipidemia and inflammation are all characteristics of a metabolic syndrome, a cluster of risk factors that is not readily applicable to the pregnant population. Nevertheless, there is evidence that maternal overweight and obesity are associated with similar derangements [27]. Thus, changes in many of the metabolic, endocrine and inflammatory parameters associated with BMI may operate as biological effectors or links between maternal adipose tissue and fetal growth and fat accretion [28]. Therefore, we analysed several factors linked to glucose and lipid metabolism at gestational weeks 30–32 a period when fetal fat accretion is high. We found a positive linear association between maternal BMI and circulating levels of fasting glucose, insulin, triglycerides, free fatty acids and a negative correlation with HDL- cholesterol (Table 3).

Plasma glucose is related to maternal nutritional state and at the same time, the principal energy supplying nutrient for fetal growth, and thus an obvious biological effector candidate. Glucose was an independent determinant of neonatal fat percentage in our study (Model 1 Table 4). This finding is in accordance with the HAPO study [8]. Fetuses are capable of de novo lipid synthesis and the excess energy supply from glucose may readily be stored as triglycerides in the fetus [9].

The fact that babies of mothers with GDM, despite strict glucose control, have increased fat mass may indicate that other metabolic parameters play a role in determining birth weight and fat accretion [13]. However, in this study, we found that the other metabolic measures were not significantly associated with neonatal fat percentage in univariate analysis, except borderline HDL- cholesterol (p = 0.049) and free fatty acids (p = 0.054). In the multivariable analysis only BMI and fasting glucose remained significant (Model 1 Table 4).

We are not aware of other studies of the association between maternal lipids and neonatal body fat, but previous studies of the role of plasma lipids in relation to birthweight have been inconsistent [11], [12].

Maternal factors may, in principle, affect fetal growth either independently of placenta, or by modifying placental nutritional transport, metabolism and endocrine functions. The transport capacity depends on total surface area, but for many compounds also on the efficacy of specific transporters [29].

It is well established that maternal BMI is related to placental size, and placental size is closely related to birthweight [30]. In the current study we also found a significant correlation between placetnal weight and neonatal fat percentage (Table 4). Taken together, it is conceivable that some of the effect of BMI on fetal fat accretion is dependent on placental size, transport capacity as well as endocrine and metabolic properties. Thus, the observed modifying effect of placental weight on the association between maternal BMI and fetal fat accretion is biologically reasonable.

### Factors Associated with Placental Weight

An emerging view is that placenta functions as a nutrient sensor, by responding to fluctuations in nutrient concentrations in the maternal circulation [13]. [13], [31]. We therefore evaluated the effect of maternal metabolic parameters on placental weight. In the univariate analyses, maternal BMI, insulin, triglycerides and total cholesterol were all positively associated with placental weight, whereas HDL-cholesterol was negatively related. No association was found between the level of free fatty acids and placental weight, while the p-value for glucose was borderline (Table 5). These findings are in accordance with previous studies and the notion of placenta as a nutrient sensor [32], [33].

In the multiple regression model, we found that BMI, gestational weight gain, parity and HDL- and total cholesterol remained independent predictors of placental weight (Table 5).

Interestingly, these independent determinants of placental weight differ from those predicting neonatal fat percentage.

Cholesterol is an integral part of all membranes. In the placenta with its large syncytial microvilli-covered surface (approximately 11 m^2^ at term), there is a great need for cholesterol for growth, renewal and steroid hormone production. HDL is a transporter of cholesterol, primarily involved in reverse transport of cholesterol, i.e. from tissues to the liver [34]. Thus, our finding of a negative association between HDL and placental size appears biologically conceivable as the level of HDL cholesterol may affect the net transport of maternal cholesterol to placenta.

The biological interpretation of the results of univariable and multiple analyses in studies like the current one needs cautious considerations given the close interrelationships between the metabolic parameters involved. For example, insulin has been shown to stimulate placental glucose transport, regulate amino acids transport (System A), lipolysis of triglycerides and possibly growth of placenta.[29], [35]–[37]. Thus, despite the absence of a significant effect of third trimester insulin on placental weight in the final multiple model, it is still conceivable that maternal insulin may play an indirect role in fetal growth.

Our study has limitations. First, this is an observational study, and therefore cannot assess causality in associations. We are aware that placental weight may be considered as a mediator rather than confounder. Accordingly, we have presented results both with and without adjustment for placenta. Possible effects of gender have not been addressed in the current study due to lack of power. However, we acknowledge the fact that newborn males and females show differences in body composition (fat-lean mass) and possible gender specific effects and effect modification by gender should be explored in future studies.

We chose not to include dietary data in the final analysis in the current paper even though maternal nutritional intake is a potential confounder in the relation between maternal BMI and neonatal fat percentage. This decision was based on preliminary analysis showing no significant effects of reported dietary intake on neonatal fat percentage (data not shown). Furthermore, the food frequency questionnaire used in the current study has not been sufficiently validated. However, based on reported dietary intakes we find it reasonable to assume a balanced diet in the cohort. Thus our findings are in line with a previous study finding diet not to be associated with placental weight or birthweight in a well nourished population [3].

We have not examined the associations between amino acids and other nutrients and placental size and fetal fat accretion [38]. Cytokines might represent another biological effector in the association between maternal BMI and placental nutrient transfer capabilities. We have previously shown that circulating levels of several cytokines are modified in overweight and obesity and other studies have shown interleukin 6 (IL-6) to be a marker of fetal adiposity and a stimulator for amino acid transport [31], [39], [40], but these and similar factors have not been considered in the current study.

Another limitation may be the use of maternal BMI as a substitute for maternal fat mass. However, in the same cohort we have performed caliper measurements and also body composition based on bioimpedance measurements. Analyses using these measurements of maternal fat mass did not substantially change the results nor did they yield additional information (data not shown). In addition maternal BMI has previously been found be reasonably well correlated with maternal fat mass [41].

Weight of placenta as a parameter of placental function needs considerations. Unfortunately, there is no specific marker that reflects overall placental transport function. However, placental weight is related to total placental surface area, and given a reasonably constant density of transporters, also to total transport capacity [42]. Accordingly, placental weight may in *normal* pregnancies be used as a substitute for functional capacity. Nutrient transport across the placenta is a complex physiological process, and dependent on the actual nutrient, the transportation process occurs by a facilitated transport down a concentration gradient (glucose) or an active transport against a concentration gradient (amino acids). Although the ratio birthweight to placental weight is a commonly used as a definition of placental efficacy, it does not take into account these different aspects of nutrient transport.

Another limitation in our study is the use of placental weight including both cord and membranes (untrimmed). However, in previous studies, trimmed and untrimmed placentas are shown to be highly correlated (Spearman’s r = 0.96) [15], [43].We acknowledge the problem that placental weight was determined at birth and not at the gestational age when the maternal blood samples were taken (beginning of last trimester). There are no reliable methods to estimate placental weight before birth, but growth of a normal placenta is close to linear which provides justification for using placental weight at birth [44].

A strength of the current study is the use of DXA, a direct and reliable method of estimating body composition in the newborns. DXA-scanning was performed close to birth (mean 1.8 day), and in a rather large population (n = 207).

### Conclusion

The associations between maternal BMI and glucose and neonatal body fat are consistent with a central role of the maternal metabolic state in nutrient and energy supply to the fetus. This effect was found to disappear by introducing placental weight as a covariate. Furthermore, several metabolic factors associated with maternal BMI were determinants of placental weight, but not of neonatal body fat.

Our findings are consistent with a concept that the effects of maternal BMI and a number of BMI-related metabolic factors on fetal fat accretion to a significant extent act by modifying placental weight.
